# Heart failure promotes gingival inflammation and impairs periodontal remodeling

**DOI:** 10.1038/s41598-026-58806-2

**Published:** 2026-07-04

**Authors:** Martin Berger, Marta Rizk, Jaana-Sophia Kern, Ulrike Schulze-Späte, Sandra Hennecke, Maximilian Neuhaus, Florian Kahles, Julia Moellmann, Stefan Wolfart, Joachim Jankowski, Michael Wolf, Katharina Marx-Schütt, Nikolaus Marx

**Affiliations:** 1 Department of Internal Medicine I, University Hospital Aachen, RWTH Aachen University, Pauwelsstraße 30, 52074 Aachen, Germany; 2 Department of Orthodontics, University Hospital Aachen, RWTH Aachen University, Pauwelsstraße 30, 52074 Aachen, Germany; 3 Department of Prosthodontics and Biomaterials, Center for Implantology, University Hospital Aachen, RWTH Aachen University, Pauwelsstraße 30, 52074 Aachen, Germany; 4 Section of Geriodontics, Department of Conservative Dentistry and Periodontology, Center of Dental Medicine, University Hospital Jena, Jena, Germany; 5 Institute for Molecular Cardiovascular Research, University Hospital Aachen, RWTH Aachen University, Pauwelsstraße 30, 52074 Aachen, Germany

**Keywords:** Cardiology, Diseases, Medical research

## Abstract

Observational studies suggest an association between impaired oral health and cardiovascular disease; however, the directionality and underlying mechanisms remain unclear. In particular, whether heart failure (HF) itself adversely affects oral and periodontal health has not been systematically investigated in large populations or experimental models.We examined the association between HF and self-reported oral health indicators in 502,387 participants of the UK Biobank, including 17,356 individuals with HF defined by ICD-9/10 codes. Multivariable logistic regression models adjusted for demographic factors, cardiovascular comorbidities, systemic inflammation, lifestyle, and socioeconomic status were applied. To explore causality and mechanisms, periodontal tissue remodeling and inflammation were assessed in a murine model of pressure overload–induced HF using transverse aortic constriction (TAC). Periodontal ligament (PDL) space and alveolar bone microarchitecture were quantified by micro-computed tomography, and gingival inflammatory gene expression was analyzed by RT–PCR.HF patients exhibited a significantly higher prevalence of oral health burden compared with controls (51% vs. 40%, p<0.001). HF was associated with a 1.6-fold increased risk of impaired oral health, which remained significant after full adjustment (adjusted OR 1.18, 95% CI 1.14–1.22; p<0.001). In mice, reduced left ventricular ejection fraction following TAC was strongly associated with expansion of the maxillary PDL space (R^2^ = 0.63, p = 0.009) and alterations in alveolar bone microarchitecture (trabecular thickness R^2^ = 0.41 p = 0.061, trabecular number R^2^ = 0.38 p = 0.07). These structural changes were accompanied by increased gingival expression of pro-inflammatory cytokines, including Il1b (SHAM vs. TAC: 1.44 ± 1.02 vs. 3.39 ± 1.53, *p* = 0.06) and TNF-α (SHAM vs. TAC: 1.37 ± 0.86 vs. 5.71 ± 1.23, *p* = 0.002 predominantly in the maxilla.HF is independently associated with impaired oral health in a large population cohort and induces site-specific periodontal inflammation and remodelling in experimental HF. These findings support HF as an upstream driver of compromised oral-periodontal health, challenging the prevailing concept that oral disease primarily contributes to cardiovascular pathology.

## Introduction

Various observational studies have suggested that impaired oral health such as periodontitis or dental caries is associated with an increased risk for the development of cardiovascular diseases (CVD) including coronary artery disease and heart failure (HF)^1,2^. Still, recent Mendelian randomisation studies have failed to demonstrate a causal effect of periodontitis on the development of heart failure or other clinical manifestations of cardiovascular disease^3,4^. In addition, first reports in a small number of heart transplant patients demonstrate that patients with HF exhibit more severe periodontal disease with elevated bone turnover and inflammation markers compared to controls, raising the hypothesis that systemic diseases such as HF impact oral health^5^. Still, hitherto the evidence of the impact of HF on oral health remains limited.

The interplay between systemic diseases and oral health is a critical area of investigation, as emerging research reveals that disorders in distant organ systems can significantly disrupt the integrity of oral soft and hard tissues^6^. Periodontal disease is characterized as a chronic inflammation of the entire periodontium, including the gingiva, periodontal ligament, cementum, and alveolar bone. Its development is multifactorial, driven by the interplay of genetic susceptibility, environmental and lifestyle factors, the oral microbiome, systemic health conditions, and host immune responses^7^. In this context, inflammatory cells and pro-inflammatory cytokines play a crucial role, as the exaggerated host immune-inflammatory response, induced by local bacterial challenge, contributes to the destruction of periodontal tissue leading to widening of the periodontal ligament (PDL) space and alveolar bone resorption^8^. The altered periodontal remodeling can ultimately lead to tooth loss^9^. Periodontitis is regarded as a local infectious disease caused by periodontopathogenic bacteria present in dental biofilm.^10^ While a dysbiotic biofilm plays an important role in the occurrence of periodontitis, recent evidence highlights the significance of systemic factors, particularly low-grade systemic inflammation, which can adversely affect the regenerative capacity of the periodontal system^11^. It has become increasingly clear that dysfunctions in other organs – such as cardiovascular disease, diabetes, or chronic kidney disease – can influence periodontal remodeling by systemic alterations of the local periodontal environment. However, while the connection between periodontitis, CVD and other diseases is well documented^12^, the nature and direction of this relationship requires further exploration^13,14^.

Previous research conducted by our group indicates that patients with heart failure (HF) exhibit more severe periodontal disease alongside elevated bone turnover markers compared to controls^5^. However, it should be noted that these findings were derived from a small cohort of HF patients who had undergone heart transplantation. Consequently, it remains uncertain whether HF in a broader population correlates with poor oral health and alterations in the periodontal system. To address this knowledge gap, the current study investigates the impact of heart failure on oral health among individuals participating in the UK Biobank and analyses the effect of HF on periodontal tissue in a murine HF model.

## Methods

### UK biobank analysis

The UK Biobank is a prospective cohort of approximately 500,000 participants enrolled between 2006 and 2010 aged between 40–69 years who lived ~25 miles from one of the 22 assessment centers located across the UK^15^. With their consent, they provided detailed information about their lifestyle, physical measures and had blood, urine and saliva samples collected and stored for future analysis. The UK Biobank cohort study obtained full ethical approval from the North West Multicentre Research Ethics Committee (10 May 2016). The study is conducted according to the Declaration of Helsinki. For this substudy, 17,356 patients with the diagnosis heart failure according to ICD-9 (4280–4289) and ICD-10 (I50.0 – I50.9) classification were selected and assessed for self-reported oral health. Use of data for this study was approved by the UK Biobank (Application number 88924).

### Assessment of oral health, cardiovascular disease and lifestyle factors

Participants completed a touchscreen questionnaire at the baseline visit, providing information on their self reported oral health indicators including mouth ulcers, painful gums, bleeding gums, loose teeth, denture treatment, and toothache. Patients were classified as having a compromised oral health status if any of these conditions were present (i.e. Composite of oral health indicators). In addition, socioeconomic status (assessed using the Townsend deprivation index), and education level (categorized as holding a university degree or not) as well as lifestyle factors, including smoking status, alcohol consumption, and food intake were collected. Furthermore, medical conditions were assessed according to ICD-9 and ICD-10 classification (i.e. majority of all patients was diagnosed according to ICD-10 classification).

### Mouse model

All animal experiments were approved by the LANUV-NRW Germany (protocol number AZ 81-02.04.2022.A162). All experiments were performed in accordance with the German legislation governing animal studies following the Guide for the Care and Use of Laboratory Animals (NIH publication, 8th edition, 2011) and the 2010/63/EU Directive on the protection of animals used for scientific purposes (Official Journal of the European Union, 2010). Male C57BL/6J mice were obtained from Janvier Labs and bred in the animal facility of the University Hospital, RWTH Aachen University, and placed with one to three animals per cage in a 12-hour day-night cycle at libitum access to food and water. At the age of 20 weeks, mice underwent transverse aortic constriction (TAC) surgery as previously described^16^ to induce pressure overload-induced cardiac hypertrophy or sham procedure as control. Mice with a body weight of 32.2 to 36.9 g were anesthetized and analgesia was applied. Mice were anesthetized with isoflurane (2%) and received preoperative analgesia with buprenorphine (0.1 mg/kg body weight) 30 minutes prior surgery. After intubation, the chest was opened by a small incision in the second intercostal space. Transverse aortic constriction was performed by tying a ligature around a 27G needle. In control mice, a sham procedure was conducted, where the thread was positioned but not tightened. All mice were sacrificed 10 weeks after surgery by cervical dislocation under isoflurane anesthesia. In total, 6 mice underwent transverse aortic constriction (TAC), while 3 mice underwent a sham procedure.

### Echocardiography measurement

Transthoracic echocardiography was performed using a Visualsonics Vevo 3100 system with MX550D transducer (Fujifilm VisualSonics) 8 weeks after surgery. Mice were placed on a heated table (37°C) and heart rate, respiration rate, and body temperature were continuously measured. Anesthesia with 1-1.5% Isoflurane was adjusted to sustain a target heart rate between 415–450 bpm. Left ventricular ejection fraction was obtained from parasternal long axis B-Mode and analyzed using VevoLab (v5.8, Fujifilm VisualSonics). All parameters were measured at least three times and means were calculated.

### PCR

PCR was conducted as previously described^17^. In brief, total ribonucleic acid (RNA) from gingiva was isolated with the RNeasy Mini Kit (#74106) (Qiagen, Hilden, Germany) and RNA preparation was followed by DNase digestion (#18068015) and reverse transcription into complementary DNA (cDNA) (#18080051) (both Thermo Fisher Scientific, Massachusetts, USA). Gene expression was quantified by use of SYBR Green reagent (#11760500) (Thermo Fisher Scientific, Massachusetts, USA) with a ViiATM 7 Real-Time PCR System (Applied Biosystems, Massachusetts, USA). Measurements were conducted in duplicates under standard reaction conditions and normalized to b-actin (Actb). Genes were selected based on their pathophysiological/physiological function (fibrosis: Tgfb and inflammation: Tnf, Il1b, Adgre1).

### Micro-computertomographie (micro-CT) assessment

Maxillae from both SHAM and TAC groups were scanned using a high-resolution micro-CT system (Skyscan 1272, Bruker Micro-CT, Belgium) at 70 kV and 130 µA with a 0.5 mm aluminum filter, an exposure time of 1870 ms and a nominal resolution of 5^3^ µm^3^. Post-reconstruction in NRecon (Bruker Micro-CT, Belgium), datasets were registered to a reference dataset in DataViewer (Bruker Micro-CT, Belgium) according to previously described methods^18^ for consistent positioning of the studied volume of interest across cohorts, focusing on the region from the first to the third molar as a reference zone. Subsequent analysis was conducted in CTAn (Bruker Micro-CT, Belgium).

Two volumes of interest (VOIs) were defined around the first molar tooth (M1): one encompassing the periodontal ligament (PDL) space and another within the alveolar bone socket of M1. After binarization based on global thresholding, PDL boundaries were delineated using seed-based segmentation for the tooth surface and a closing algorithm for the alveolar bone at the PDL-bone interface. The PDL space was defined as the region with x-ray opacity below that of alveolar bone or dentin, located between tooth surface and alveolar bone periphery, with PDL thickness determined via algorithms for trabecular thickness. For alveolar bone analysis, teeth were segmented out from VOIs, followed by virtual closure of bone peripheries to assess morphology through measurements of trabecular thickness (Tr. Thickness) and trabecular number per section (Tr. Number). Data normality was assessed using GraphPad Prism (version 10.3.1), and correlations with left ventricular ejection fraction were evaluated.

### Statistical analysis

The baseline characteristics are reported as means with standard deviations in case of continuous variables, and frequencies with percentages in case of binary variables. For comparison between two groups, categorical variables were analyzed using the chi-square test, while continuous variables were compared using Student’s t-test or the Mann–Whitney U test, as appropriate. For logistic regression models, continuous variables were tested for normal distribution using the Kolmogorov–Smirnov test, visually assessed by histograms and log transformed before analysis when required. Odds ratios (HRs) and 95% confidence intervals (95% CIs) were calculated and adjusted for potential confounders as indicated. In a basic model (model 1), we adjusted for age and sex. In a second model (model 2), we additionally adjusted coronary artery disease (CAD), presence of diabetes (according to ICD), and presence of hypertension (according to ICD). Next, we additionally adjusted for hsCRP. In a final model we adjusted for tobacco use, food intake, academic qualifications, income, and alcohol use (model 5). All statistical tests were 2-sided, and a p-value <0.05 was considered significant. The analyses were performed using R version v. 4.2.2 and Graphpad Prism v10.

## Results

### Association of heart failure with oral health in the UK biobank

Data on perceived oral health were available of 502,387 individuals in the UK Biobank, 17,356 of whom had been diagnosed with heart failure according to ICD-9 and ICD-10. Patients with heart failure had a significant reduced LV-EF compared to patients without heart failure (48±11% vs. 56±7% p < 0.001). Compared to controls, patients with heart failure were older and more likely to have diabetes, coronary artery disease, and hypertension. Moreover, these patients exhibited significantly higher hs-CRP levels (4.06 vs. 2.55 mg/L [p˂0.05]) as well as a lower eGFR (83 vs. 91 [p˂0.01]) (Table 1).

**Table 1:** Characteristics of individuals according to the presence of heart failure.

	Heartfailure			
	Overall N = 502,387	Absent N = 485,031	Present N = 17,356	p-value
**Characteristic**				
Age (yrs)	57 (8)	56 (8)	62 (6)	<0.001
Weight (kg)	78 (16)	78 (16)	86 (19)	<0.001
Sex (Male)	273,311 (54%)	267,088 (55%)	6,223 (36%)	<0.001
**Patient history**				
Diabetes	21,719(4.3%)	19,015(3.9%)	2,704(16%)	<0.001
CAD	23,702(4.7%)	18,905(3.9%)	4,797(28%)	<0.001
Hypertension	135,733(27%)	126,570(26%)	9,163(53%)	<0.001
Stroke	7,667(1.5%)	6,604(1.4%)	1,063(6.1%)	<0.001
**MRI Parameters**				
LV-EF (by MRI)	55 (7)	56 (7)	48 (11)	<0.001
LVEDV (by MRI)	141 (111)	140 (80)	201 (703)	0,056
LVESV (by MRI)	64 (66)	64 (65)	96 (107)	<0.001
LVSV (by MRI)	76 (74)	76 (24)	105 (635)	0,32
**Biomarkers**				
Interleukin-6 (OLINK)^1^	0.13 (0.90)	0.10 (0.88)	0.68 (0.97)	<0.001
hsCRP (OLINK)^1^	2.60 (4.36)	2.55 (4.28)	4.06 (6.08)	<0.001
NTproBNP (OLINK)^1^	0.10 (1.26)	0.03 (1.18)	1.57 (1.85)	<0.001
HbA1c (%)	5.46 (0.62)	5.44 (0.60)	5.84 (1.03)	<0.001
Creatinine (mg/dL)	0.82 (0.21)	0.81 (0.19)	0.93 (0.47)	<0.001
eGFR (ml/min)	91 (13)	91 (13)	83 (17)	<0.001
UACR (mg/g)	30 (153)	27 (139)	78 (301)	<0.001
**Endpoints**				
All cause Mortality	37,731(7.5%)	31,748(6.5%)	5,983(34%)	<0.001
CV-Death	16,656(3.3%)	11,770(2.4%)	4,886(28%)	<0.001

^1^OLINK Dataset available for 51296 patients, data presented as normalized protein expression.

**Fig. 1:**
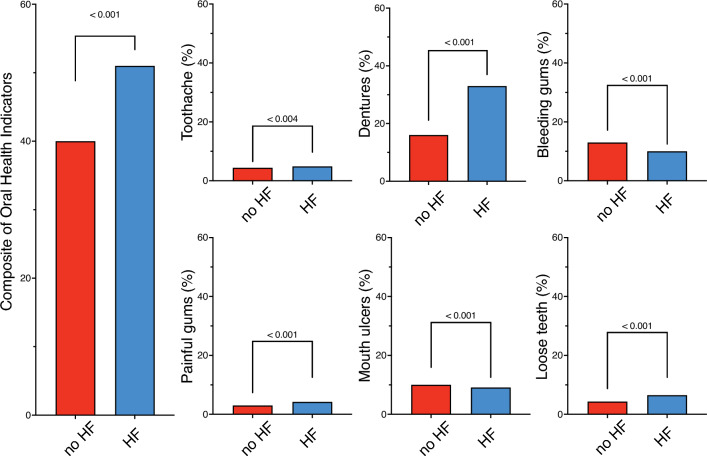
Association between heart failure (HF) and oral health status Quantification of oral health status parameters stratified by the absence (blue) or presence (red) of heart failure (HF).

The composite of perceived oral health was more prevalent among HF patients (51% vs. 40% [p˂0.001]), with the presence of dentures being the most common indicator for a compromised oral health (33% vs. 16% [p˂0.001]). Furthermore, indicators for periodontal disease such as loose teeth (6.5% vs. 4.3% [p < 0.001]) and painful gums (4.2% vs. 3% [p<0.001]) were significantly higher in HF patients (Fig. 1).

**Fig. 2:**
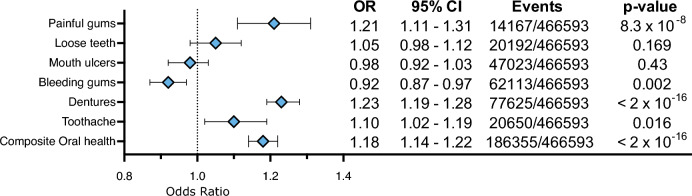
Logistic regression for the association between heart failure and self reported indicators oral health. Forest plot showing odds ratios (ORs) with 95% confidence intervals (CIs) for the association between individual oral health indicators and prevalence of heart failure. Models are based on logistic regression analyses and are adjusted for age, sex, coronary artery disease, diabetes, hypertension, hsCRP, smoking, diet, education, income and alcohol use.

The presence of heart failure was significantly associated with the composite oral health endpoint (OR 1.18 [95% CI 1.14–1.22]) after full adjustment for CAD, cardiovascular risk factors, hsCRP, and socioeconomic variables. This association was primarily driven by painful gums (OR 1.21 [95% CI 1.11–1.31]), denture use (OR 1.23 [95% CI 1.19–1.28]), and toothache (OR 1.10 [95% CI 1.02–1.19]). The only marker showing an inverse association with heart failure was bleeding gums (OR 0.92 [95% CI 0.87–0.97] Fig. 2).

### Impact of heart failure on periodontal tissue health in mice

**Fig. 3:**
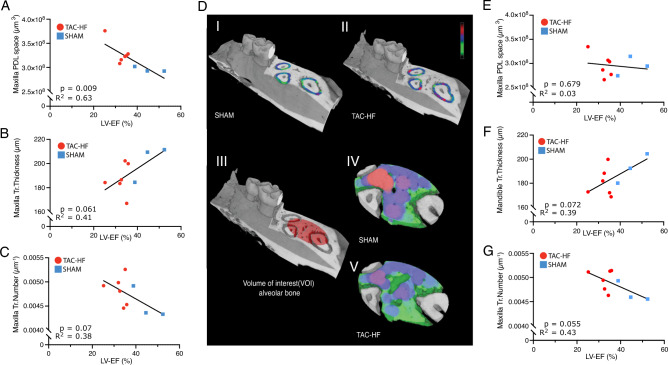
Association of impaired cardiac function with periodontal health in a mouse model of heart failure (TAC-induced HF 10 weeks after surgery versus sham operated mice). (**A**) Correlation between LV-EF and accrued PDL space volume (SHAM - blue squares, TAC-HF – red circles) for the maxillary alveolar bone. (**B,C**) Correlation between LV-EF and Trabecular thickness (**B**) or trabecular number (Tr.Number) (**C**) for the maxillary alveolar bone. (**D**) Representative 3D reconstructions of micro-CT scans of the maxilla (region of the 1^st^ up to 3^rd^ molar tooth) in SHAM (DI) mice and TAC-HF (DII) mice. Thickness of the PDL space of the 1^st^ molar tooth (M1) is color-coded, ranging from thinner regions (green), through moderate thickness (blue), to the thickest areas (red). Morphological assessment of alveolar bone within the M1 socket of the maxilla (volume of interest, red area DIII) in SHAM mice (DIV) and TAC-HF mice (DV). Trabecular thickness (Tr.Thickness) is color coded from thinner trabeculae (green), through moderate trabeculae (blue), to the thickest trabeculae (red). (**E**) Correlation between LV-EF and accrued PDL space volume (SHAM - blue squares, TAC-HF – red circles) for the mandibular alveolar bone. (**F,G**) Correlation between LV-EF and Trabecular thickness (**F**) or trabecular number (Tr.Number) (**G**) for the mandibular alveolar bone.

**Fig. 4:**
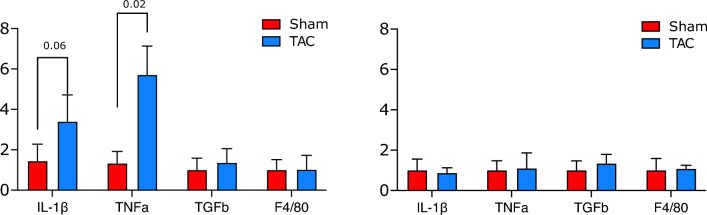
Adverse induction of inflammatory marker in the gingiva of the maxilla and mandible Quantitative real-time PCR analysis of inflammatory and macrophage-associated marker gene expression in maxillary (left panel) and mandibular (right panel) tissues from sham-operated mice (Sham, red) and mice subjected to transverse aortic constriction (TAC, blue). mRNA expression levels of *Il1b*, *Tnfa*, *Tgfb*, and *Adgre1* (F4/80) are shown, normalized to *Actb*. Data are presented as mean ± SEM and analyzed by a two-tailed t-test or Mann-Whitney U test. Sham n=2-3 and TAC: n=5-7 animals.

To further analyze to what extent the development of HF may affect periodontal tissue homeostasis and health, we employed the well-established TAC-induced HF mouse model and assessed periodontal bone structure and micro architecture by micro-CT. As shown in Fig. 3, worsening left-ventricular ejection fraction 10 weeks after TAC surgery was significantly associated with expansion of the periodontal ligament (PDL) space of the maxilla (R^2^=0.6393; p=0.0097) but not the mandible (R^2^=0.03; p=0.679). Moreover, there was a trend towards a lower trabecular thickness (Tr. Thickness) and density of trabeculae per virtual section (Tr. Number) that was similar among maxillary and mandibular bone and indicates an altered bone structure in TAC-HF mice (Fig. 4).

To mechanistically investigate alterations in the maxilla and mandible of TAC mice, we assessed the inflammatory status of the gingival tissue by RT–PCR. Notably, gingiva from the maxilla of TAC mice exhibited a trend towards increased gene expression of the pro-inflammatory cytokines IL-1β (SHAM vs. TAC: 1.44 ± 1.02 vs. 3.39 ± 1.53, *p* = 0.06) and significant increased expression of TNF-α (SHAM vs. TAC: 1.37 ± 0.86 vs. 5.71 ± 1.23, *p* = 0.002). In contrast, no inflammatory phenotype was observed in gingival tissue derived from the mandible.

## Discussion

Our patient data from the UK Biobank a as well as our experimental results in a murine model of HF suggest that HF and HF-associated oral inflammation might be a risk factor for compromised oral health.

Recent studies suggest that systemic inflammation as well as local factors influencing the local host response might be of critical importance in periodontitis and oral health. Previous data in a selected population of HF patients after heart transplantation demonstrate that patients with HF exhibit more severe periodontal disease compared to control patients^5^. Furthermore, recent Korean studies have not only concluded that dental diseases and impaired oral health are associated with heart failure among patients with type 2 diabetes but have also suggested a correlation between gingivitis, tooth loss, and increased risks of stroke and cardiovascular disease^19^.Our study now extends these data by demonstrating a strong association of self-reported oral health indicators in patients with HF. Even after multiple adjustments for potential confounders, HF remained strongly and highly significantly associated with compromised oral health showing an 18% increase in risk.

While our data supports an association between HF and self-reported oral health, the underlying mechanisms remain insufficiently understood. Interestingly, the strong association of painful gums, denture use, and loose teeth with heart failure, as well as their increased prevalence, may indicate periodontal remodeling disturbances in the context of systemic disease.

Current concepts in periodontitis emphasize the importance of systemic inflammation as well as local factors shaping the host response and periodontal tissue stability. In this context, HF-associated systemic inflammation, altered perfusion, and metabolic disturbances may adversely affect periodontal tissue homeostasis^20,21^.

Indeed, this notion is further supported by our experimental data in a murine model of HF, which showed that a reduced left ventricular ejection fraction was significantly associated with expansion of the periodontal ligament (PDL) space, suggesting a potential causal relationship between the development of HF and impaired periodontal tissue stability.

Given the known site-specific differences in jaw bone biology, including lower bone mineral density, a higher proportion of trabecular bone, and distinct vascularization of the maxillary alveolar bone, particularly in posterior regions, the maxilla may be more susceptible to systemic circulatory and inflammatory disturbances characteristic of HF^22–24^. This relative structural vulnerability, together with differences in remodeling dynamics, may contribute to the site-specific periodontal alterations observed in our murine model.

In addition, we demonstrate that HF is associated with gingival inflammation characterized by increased gene expression of pro-inflammatory cytokine TNF-α and a trend in increased IL-1β-expression. Chronic HF may therefore constitute an important driver of sustained gingival inflammation which then makes the tissue more vulnerable to pathological bacterial triggers and may result in pronounced destruction of the periodontal ligament and alveolar bone with subsequent progression from gingivitis to periodontitis^25^. Therefore, heart failure and heart failure–associated inflammation may act as upstream drivers of impaired oral health, challenging the prevailing view that oral disease primarily contributes to cardiovascular pathology.

Our study has certain limitations. Even though the overall ejection fraction in individuals with heart failure was lower compared to controls, the design of the UK Biobank does not allow differentiation between HF with reduced and preserved ejection fraction (HFrEF, HFpEF). This limitation may impact the interpretation of our findings, as these subtypes differ in their pathophysiology, risk factors, and clinical outcomes. Moreover, oral health status was self-reported without clinical validation. While individuals can assess their oral health to some extent, the questionnaire was broad and may have lacked the sensitivity to capture the full range of oral health conditions. It included indicators such as painful or bleeding gums, loose teeth, denture use, and tooth ache but these self-reports cannot distinguish between etiologies. In addition, while the UK Biobank predominantly included generally healthy individuals, patients with a substantial chronic disease burden may have underreported oral health problems due to the overall burden imposed by their disease. Our experimental data were limited to a single 10-week timepoint. Although the 10-week timepoint was carefully selected and captures the development of both periodontal disease and heart failure, we do not have long-term TAC data and therefore cannot draw conclusions beyond this timepoint. Despite these limitations, summarizing the responses as “self-reported oral health” is reasonable and has been used in prior UK Biobank studies to investigate associations with mental and cardiovascular health^26,27^.

## Conclusions

In conclusion, our analyses from the UK Biobank cohort as well as our experimental data suggest that HF might be a risk factor for compromised oral health. This data challenges the prevailing view that oral disease primarily contributes to cardiovascular pathology and deserves further attention.

## Data Availability

The data used in this study were obtained from UK Biobank under a material transfer and data access agreement. In accordance with UK Biobank’s governance framework, researchers are not permitted to share individual-level UK Biobank data with third parties. Access to the data is restricted to approved researchers and specific approved projects, and all secondary use requires a separate application directly to UK Biobank. Requests to access these datasets should be directed to the corresponding author.
